# It Is Not a Boerhaave! A Case of Spontaneous Pneumothorax

**DOI:** 10.7759/cureus.25191

**Published:** 2022-05-21

**Authors:** Imran Khan, Rukma R Govindu, Hussam Ammar

**Affiliations:** 1 Internal Medicine, MedStar Washington Hospital Center, Washington, USA; 2 Internal Medicine, The University of Texas Health Science Center at Houston, Houston, USA; 3 Internal Medicine, The University of Texas McGovern Medical School at Houston, Houston, USA

**Keywords:** diabetic gastroparesis, boerhaave's syndrome, persistent vomiting, esophageal rupture, spontaneous pneumothorax

## Abstract

Spontaneous pneumothorax is a pneumothorax that is not caused by trauma or an apparent precipitating factor. This report presents a case of a 91-year-old man with no history of lung disease who developed pneumothorax after two days of persistent nausea and vomiting. He was misdiagnosed as a case of Boerhaave’s syndrome. A chest computed tomography with iohexol oral contrast showed no evidence of esophageal rupture, and an upper endoscopy revealed a small gastric ulcer and no gastric outlet obstruction. The patient was managed conservatively; his spontaneous pneumothorax, nausea, and vomiting resolved.

## Introduction

The French physician Jean Itard was the first to describe and use the word pneumothorax in his dissertation on the subject in 1803 [1]. Pneumothorax is classified as primary spontaneous pneumothorax (PSP), secondary spontaneous pneumothorax (SSP), or traumatic pneumothorax. PSP occurs in patients with no apparent lung disease, while SSP is diagnosed in patients with pre-existing lung pathology. Both diagnoses vary in severity, outcome, and treatment plan [1-3]. PSP has a yearly incidence of 18-28 per 100,000 men and 1.2-6.0 per 100,000 population in women. The annual incidence of SSP is 6.3 per 100,000 in men and 2.0 per 100,000 in women [2].

## Case presentation

**Figure 1:**
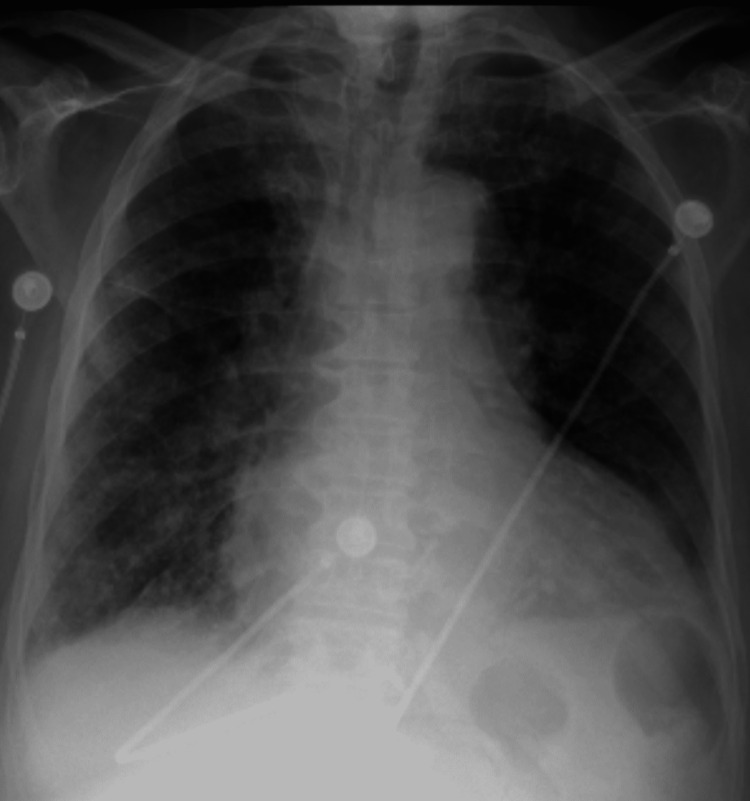
Admission chest radiography with no pneumothorax

**Figure 2:**
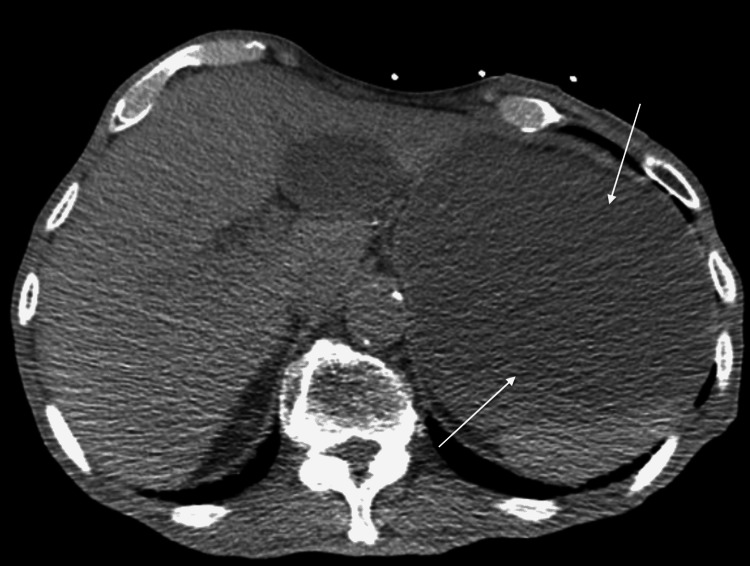
Dilated stomach "arrows" on admission computed tomography

**Figure 3:**
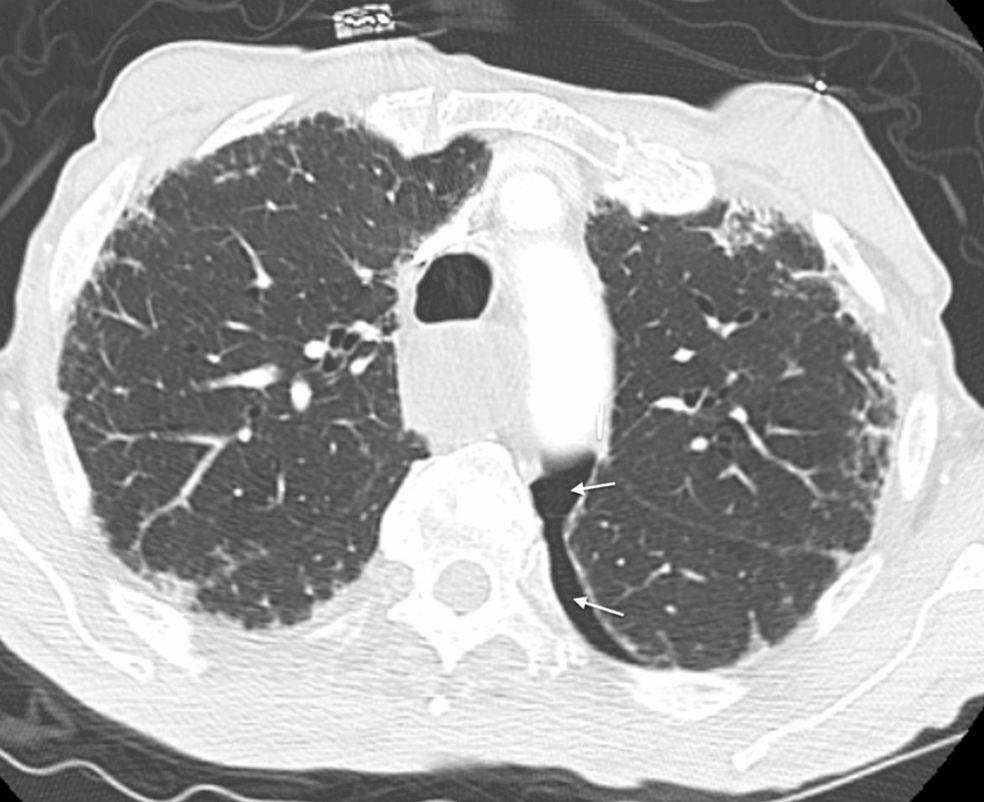
Left pneumothorax "arrows" on admission computed tomography

A 91-year-old man was transferred for evaluation of suspected esophageal rupture. He presented to an outside hospital for two days with persistent non-bilious vomiting and chest and epigastric pain. He has a 90-pack-year history of smoking and long-standing diabetes. On admission, his heart rate was 110 beats per minute, blood pressure was 160/90, his respiratory rate was 18 breaths per minute, and his temperature was 36.5 ºC. A nasogastric tube was placed at the outside facility. On examination, auscultation of the lungs revealed bilateral basal crackles, there was no subcutaneous emphysema, and the abdomen was soft and non-distended. Chest and abdomen computed tomography (CT) from the outside hospital revealed a marked gastric distention and a small left pneumothorax that was not seen on chest radiographs (Figures 1-3). A repeat CT of the chest and abdomen with oral contrast showed no evidence of esophageal rupture. Admission laboratory values were blood glucose level was 440 mg/dL, blood urea nitrogen was 19 mg/dL, creatinine 0.87 mg/dL, bicarbonate level 28 mmol/L, and blood ketone levels were negative. The white blood cell count was 14.7 x10^3^/mm^3^, and hemoglobin was 14.2 g/dL. The upper endoscopy revealed a small gastric ulcer and no gastric outlet obstruction. He never complained of dyspnea or chest pain, and his follow-up chest radiography remained without pneumothorax. The patient improved on conservative management, bowel rest, intravenous hydration, insulin, metoclopramide, pantoprazole, and eventually tolerated diet. He was discharged on day 6 with a diagnosis of spontaneous pneumothorax and intractable vomiting secondary to suspected diabetic gastroparesis. He was lost for follow-up and was not seen in our clinics.

## Discussion

The Dutch physician Hermann Boerhaave described the first case of a spontaneous esophageal rupture in 1724. Boerhaave was asked to evaluate Baron John van Wassenar who went on three days of a gluttonous feast followed by a trial of regurgitating his food, after which he felt instantaneous excruciating pain. Boerhaave, unfortunately, could not save the Baron, but he described to the world the findings of the first autopsy on a patient with spontaneous esophageal rupture [4]. The description of Boerhaave's syndrome evolved in the 21st century; in a case series of 14 patients with esophageal rupture, the classic triad of vomiting, chest pain, and subcutaneous emphysema were present in only one out of 14 patients [5]. Abdominal pain and tenderness obscured the clinical picture. Pneumomediastinum, pneumothorax, and hydropneumothorax are radiological findings in patients with Boerhaave's syndrome. Boerhaave syndrome can rarely present with pneumothorax without pneumomediastinum. Mortality rates of Boerhaave’s syndrome range from 0% if the diagnosis was made within 24 hours to 29% if it was diagnosed after 48 hours. Computed tomography with oral contrast is the diagnostic test of choice to confirm esophageal perforation [5,6].

Spontaneous pneumothorax occurs in patients with or without underlying respiratory disease and is classified as primary or secondary. PSP occurs in young and healthy persons without known lung disease. SSP is a complication of underlying lung disease (e.g., chronic obstructive pulmonary disease, tuberculosis, lung cancer, etc.). The complications of a pneumothorax are significantly more in patients with pre-existing lung disease, and the treatment is potentially more problematic. Tobacco smoking is the most important risk factor for PSP. About 88% of the patients with PSP were tobacco smokers in a retrospective Scandinavian study. Cannabis smoking can cause pneumothorax; it causes direct parenchymal damage, and the Valsalva maneuvers related to smoking cannabis may cause pulmonary barotrauma, alveolar rupture, and air leak [2,3,7]. PSP is not typically associated with known lung disease; however, most affected patients have unrecognized lung abnormalities that may predispose them to pneumothorax. Subpleural bullae and emphysema-like changes (ELCs) were identified on computed tomography and during thoracotomy in patients with primary pneumothorax. There is no definitive evidence that ELCs are the unique cause of pneumothorax. Smoking provokes small airway disease; the narrowed inflamed small airway disease can cause air trapping distal to the inflamed airways and higher pressure difference from barotrauma can cause an alveolar rupture with an air leak and pneumothorax. Pneumothorax may be entirely asymptomatic as in this case and can be seen as an incidental finding on radiological images. It usually causes an abrupt onset of chest pain, dyspnea, and sometimes cough. The severity of the symptoms depends on the presence or absence of underlying lung disease and the severity of pneumothorax. [2,3,7].

Pneumothorax is diagnosed in most patients by inspiratory chest radiography. CT is the gold standard test; it can detect small pneumothorax that cannot be seen on chest radiography. CT can also identify the presence of large bullae that occur in patients with emphysema and can mimic the radiological appearance of pneumothorax on chest radiography because of the absence of pulmonary markings within the bulla. Small asymptomatic pneumothorax (≤ 2 cm at the hilum or ≤ 3 cm at the lung apex) is managed conservatively with observation, while large or symptomatic pneumothorax requires chest drain or small-bore catheter. Both Primary and secondary pneumothorax tend to recur; the recurrence rate is higher in secondary pneumothorax as 40%-50% of SPP cases will recur [2,3,7].

The patient, in this case, has probably undiagnosed chronic obstructive pulmonary disease after decades of smoking. His spontaneous pneumothorax is probably SSP. Cases of spontaneous pneumomediastinum and spontaneous pneumothorax secondary to pulmonary barotrauma from increased intrathoracic pressure and alveolar rupture by intractable vomiting in diabetic ketoacidosis, hyperemesis gravidarum, and marijuana smoking have been reported [8].

## Conclusions

Persistent vomiting and retching can rarely cause isolated spontaneous pneumothorax. The clinical presentation of spontaneous pneumothorax after persistent vomiting cannot be precisely differentiated from Boerhaave's syndrome, a potentially fatal condition based on chest radiography and physical exam. It is imperative to perform an imaging evaluation of the esophagus with an oral contrast study to rule out esophageal perforation.
